# Telomeres are elongated in older individuals in a hibernating rodent, the edible dormouse (*Glis glis*)

**DOI:** 10.1038/srep36856

**Published:** 2016-11-24

**Authors:** Franz Hoelzl, Steve Smith, Jessica S. Cornils, Denise Aydinonat, Claudia Bieber, Thomas Ruf

**Affiliations:** 1 Department of Integrative Biology and Evolution, University of Veterinary Medicine, Vienna, Austria

## Abstract

Telomere shortening is thought to be an important biomarker for life history traits such as lifespan and aging, and can be indicative of genome integrity, survival probability and the risk of cancer development. In humans and other animals, telomeres almost always shorten with age, with more rapid telomere attrition in short-lived species. Here, we show that in the edible dormouse (*Glis glis*) telomere length significantly increases from an age of 6 to an age of 9 years. While this finding could be due to higher survival of individuals with longer telomeres, we also found, using longitudinal measurements, a positive effect of age on the rate of telomere elongation within older individuals. To our knowledge, no previous study has reported such an effect of age on telomere lengthening. We attribute this exceptional pattern to the peculiar life-history of this species, which skips reproduction in years with low food availability. Further, we show that this “sit tight” strategy in the timing of reproduction is associated with an increasing likelihood for an individual to reproduce as it ages. As reproduction could facilitate telomere attrition, this life-history strategy may have led to the evolution of increased somatic maintenance and telomere elongation with increasing age.

Telomeres, which in vertebrates contain many tandem repeats of the sequence motif 5′-TTAGGG-3′^1^, are the endcaps of chromosomes which prevent, together with telomere associated proteins (the sheltering complex), the degradation of coding DNA sequences. In normal somatic cells, telomeres are shortened with every cell division – due to the end replication problem in mitosis^2^,^3^. In addition to this shortening during cell proliferation, oxidative stress has a strong effect on telomere erosion^4^. Reactive oxygen species (ROS), which are a by-product of mitochondrial respiration, can adversely affect chromosomal DNA by oxidizing nucleotides. ROS induced telomere loss could also explain why telomere loss is much higher (50–200 base pairs [bp] per cell division) than suggested by the telomere loss which occurs due to the end replication problem (~10 bp per cell division)^5^,^6^,^7^. However, the rate of telomere shortening differs between species. For instance, it has been shown that telomeres in fast-aging, short-lived wild animals shorten faster than in slow-aging, long-lived ones^8^,^9^.

Telomere length can be maintained by the enzyme telomerase^10^ or by DNA-recombination, known as alternative lengthening of telomeres (ALT)^11^. It is also known that telomere length is not only maintained but can also be increased by these mechanisms (in somatic and germline cells). For instance, in two species of hibernating dormice, telomere length is shortened over the hibernation season during bouts of rewarming^12^,^13^,^14^, which are associated with increased oxidative stress^15^,^16^. However, for adult edible dormice it was shown that telomeres are restored and even increase in length during the summer active season^12^,^14^.

This capability of edible dormice to re-elongate telomeres runs contrary to the accepted process of progressive, systematic shortening of telomeres with age and raises questions about the long-term balance between telomere attrition and repair. To test if telomere length decreases, is maintained, or even increases with progressing age we determined relative telomere length (RTL) as well as changes of RTL over time by recapturing and repeatedly collecting mucosa samples from free-living dormice (2–5 times per individual) aged 1–9 years from 2012–2014. Since edible dormice, similar to other hibernators^17^ are long-lived and can reach a lifespan of 13 years in the wild^18^, we hypothesized that they would show high levels of investment into somatic maintenance and hence slow shortening or even stabilised levels of RTL.

The high longevity of edible dormice is partly due to their skipping of reproduction in years of tree-seeding failure (a major food resource for juveniles), which leads to a large variation in yearly reproductive effort^19^. As reproduction is potentially associated with oxidative stress and telomere attrition, we recorded reproduction (in females) and reproductive capability (in males) in the same study population in which we monitored RTL. The rationale behind this approach was that telomeres might provide a marker of the costs of reproduction, which is accessible even in field studies. We expected that reproduction may exacerbate telomere attrition and that, vice versa, high levels of telomere maintenance should be reflected by slow or absent reproductive senescence.

## Results

**Figure 1:**
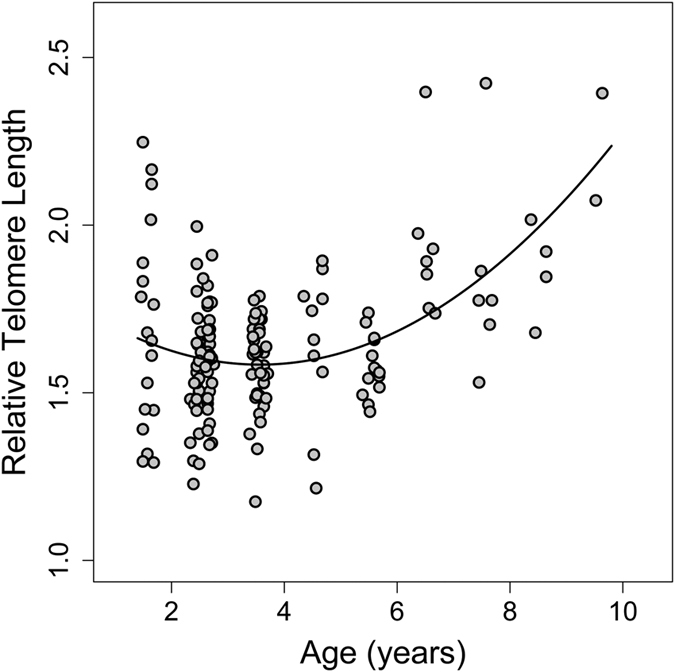
Effect of age on relative telomere length (RTL) in edible dormice (n = 49, N = 158). The effect of age was best described by a quadratic polynomial (age coefficient −0.14, SE 0.07; P = 0.033; age^2^ coefficient 0.019; SE 0.007; P = 0.005). Component and residuals plot from a linear mixed effects model. The r^2^ of the full model was 0.53; the r^2^ of random effects was 0.40.

Among the variables tested, only age significantly affected RTL (Fig. 1) in a non-linear pattern with RTL decreasing in younger (≤~5 years) and increasing in older dormice. RTL was not affected by time of the year, sex, body mass or reproductive activity/capability at the time of sampling. The effect of reproductive activity also remained non-significant when the model was restricted to females only.

**Figure 2:**
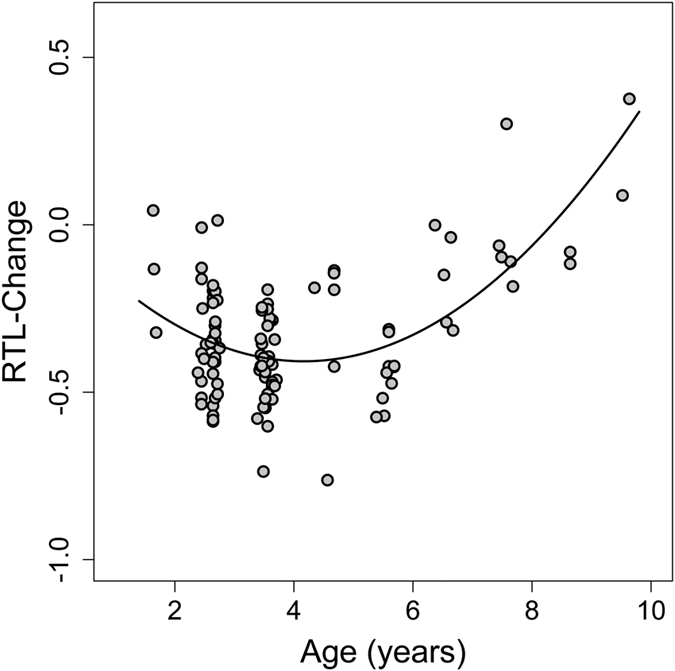
Effect of age on the change of relative telomere length (RTL) in edible dormice (n = 49, N = 109). The effect of age was best described by a quadratic polynomial (age coefficient −0.18, SE 0.08; P = 0.020; age^2^ coefficient 0.022; SE 0.007; P = 0.003). Component and residuals plot from a linear mixed effects model. The r^2^ of the full model was 0.62; the r^2^ of random effect was 0.43.

Among all variables tested, once again only age had a significant nonlinear effect on RTL-change (Fig. 2), confirming the pattern observed for RTL as such.

**Figure 3:**
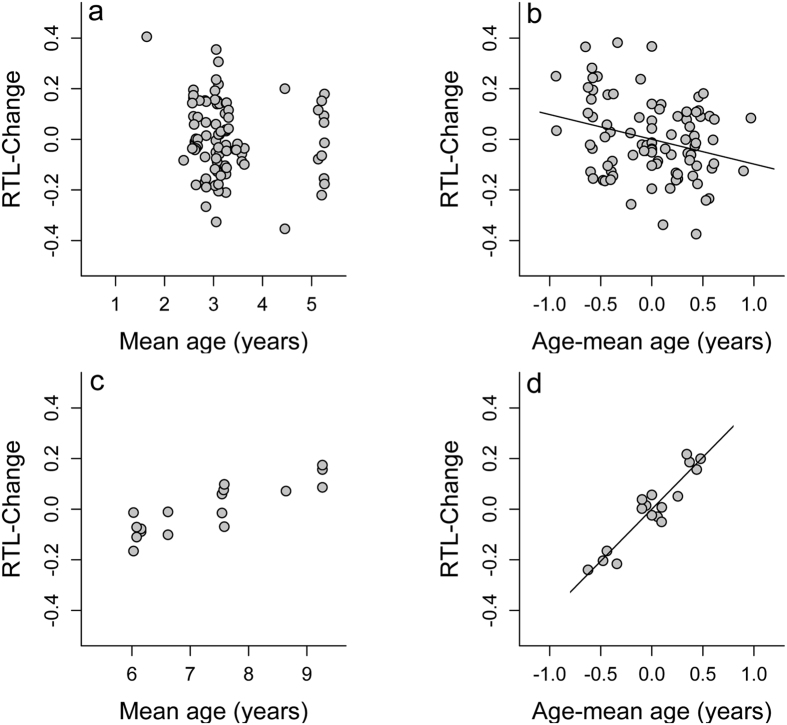
Differences in RTL changes among younger (panels **a**,**b**) and older dormice (panels **c**,**d**). There were no significant between-subject effects in either age group (panels **a**,**c**). There was, however, significant RTL shortening within younger dormice (**b**) and significant RTL elongation within older dormice (**d**) Age groups were split at a mean age of 5.3 years, which minimized the combined sum of squares.

Separating between-subjects and within-subjects effects on RTL as such gave no evidence for significant between-subjects or within-subjects effects. However, after adjusting for variation in initial telomere length, we found that RTL-change within individuals was age dependent (Fig. 3). Up to an age of 5.3 years, RTL-change was predominantly negative (shortening of telomeres; slope = −0.097; SE = 0.042; P = 0.028, Fig. 3b), whereas older individuals significantly elongated RTL (slope = +0.410; SE = 0.066; P = 0.003, Fig. 3d).

**Figure 4:**
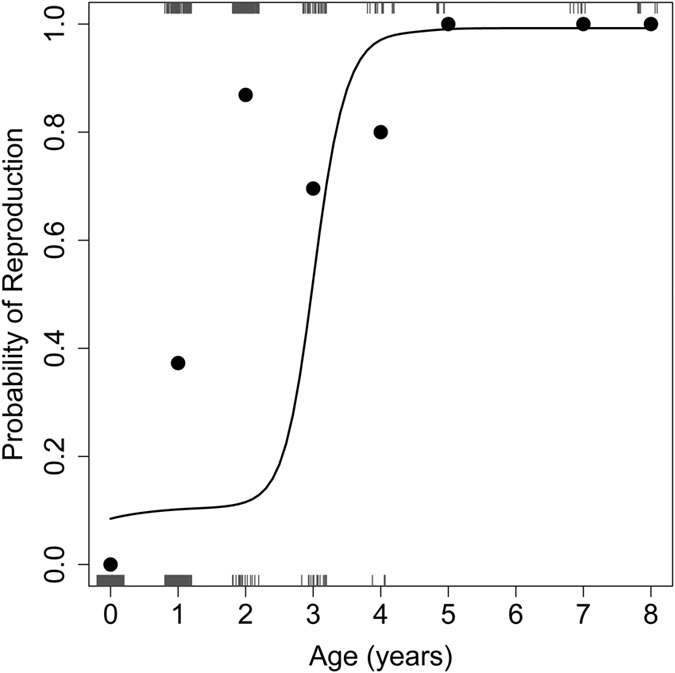
Effect of age on the probability of female dormice to reproduce (n = 1529, N = 816). Black circles show means for each age. The sigmoidal prediction line was obtained from a mixed effects logistic regression and was averaged over all observation years and individuals. The vertical lines close to the upper and lower x-axes each indicate an observation of reproduction (1) or no reproduction (0), respectively.

Age positively affected the probability of females to reproduce (Chi^2^ = 98.8, P < 0.001; n = 1529). The likelihood to reproduce in any given year reached 1 at an age of 5 years and remained at this level up to the oldest age observed (Fig. 4). There was however, no detectable effect of age on litter size (Chi^2^ = 1.2, P = 0.28, n = 115). Mean litter size was 5.49 ± 2.14 SD.

## Discussion

Surprisingly, and contrary to our hypothesis, telomeres were shortened only in younger dormice but were elongated in older animals. To our knowledge, no other longitudinal study on humans or other animals has reported such an effect. A number of previous studies found evidence for the opposite, an association between telomere shortening and aging^9^,^20^,^21^,^22^. It has been shown by Hoelzl *et al*.^14^ and Turbill *et al*.^12^ that telomeres in edible dormice can be elongated, but these increases were not associated with age. Another study in planarians (invertebrates) also showed that telomeres can be elongated but again without an age effect^23^. In vertebrates, there has been only one other study, on Leach’s storm petrels (*Oceanodroma leucorhoa*), that has suggested telomere elongation or at least slowed down telomere shortening as explanations for their detection of longer telomeres in older animals^24^. However, as this was a cross-sectional study, this observation could have been caused by higher survival of birds that had longer telomeres as juveniles. In contrast, here we also show that, in edible dormice, not only RTL as such (Fig. 1), but also the change of telomere length within individuals systematically changed with age (Fig. 2). While young animals typically showed RTL shortening, older animals predominantly elongated telomeres (Fig. 3). A possible decrease in telomere attrition over time, when constant telomerase activity is assumed, an increase in telomere elongation, or a combination of both effects, seem the most parsimonious explanations for the observed increase in absolute RTL with age in older individuals. However, we cannot rule out the possibility of selective survival of individuals that were particularly able to elongate telomeres. Even if that was the case, however, our data still demonstrate that RTL in older dormice is not just the result of initial telomere length in young animals and inevitable telomere shortening, but is also affected by telomere elongation.

An interesting question raised by this finding is what fitness consequences are implied by longer telomeres, and further, why elongating telomeres over time could be beneficial for older edible dormice. One might expect that animals maintain telomeres on a relatively long and constant length to retain a high potential of cell renewal. Also, assuming that RTL determined in buccal mucosa cells are representative for all somatic cells, high stable amounts of telomeric DNA may indicate reduced aging, e.g. via low cell turnover rates. This would help to explain why edible dormice reach a maximum lifespan of up to 13 years^18^, but it remains an intriguing question why telomeres should actually be elongated over time.

It is known that in both edible and garden dormice telomeres are significantly shortened over the hibernation season, especially during intermittent periods of rewarming to euthermia^12^,^13^,^14^. Thus, one could expect that dormice restore telomere length after hibernation by means of telomerase or ALT. Telomere restoration aside, an increase in telomere elongation at older age in order to anticipate and counteract the negative impact of the next hibernation season should only be expected if there is also an age-related increase in hibernation duration, or the time spent at euthermia during the winter. However, long-term (14 years) records of dormice (n = 75; aged 1 to 10 years) in outdoor enclosures, including continuous over-winter body temperature records (n = 33 data sets), provide no evidence for such an effect of age (CB & TR, unpublished).

Another physiological function that may be associated with increased oxidative stress and telomere loss is reproduction. However, clear evidence for reproduction-related oxidative damage mainly comes from domesticated livestock, that is, animals selected for extreme reproductive output (review in Metcalf and Monaghan^25^). We hypothesized that edible dormice may be a good candidate species to detect reproduction-related oxidative damage in a wild animal. This is because lactating females reach very high rates of energy turnover (>6 x basal metabolic rate)^19^ even when fed *ad libitum* (with minimum foraging costs) and dormouse pups grow extremely fast during lactation^26^. Our analysis gave no evidence, however, for an effect of reproductive activity at the DNA sampling time on cellular damage in terms of either absolute RTL or RTL change.

It would be premature, however, to use this lack of a statistical effect to dismiss the possibility of effects of reproduction on telomere length, as several other studies found an effect of reproduction on telomere length^27^,^28^,^29^. This is because RTL in our study was determined at random times over the active season, whenever the animals were encountered. Directly testing for telomere attrition during reproductive bouts would require determining RTL immediately prior to and following reproduction in the same animals, which was not feasible here. Therefore, the fact that, apart from RTL, the probability to reproduce in any given year was the only trait that also increased with age in dormice (Fig. 4) remains intriguing. Since older animals are more likely to reproduce, arguably due to diminishing prospects of future survival, it may well be adaptive for them to preventively elongate telomere length. This is because the most common (50%) nucleobase in telomeres is Guanine, which is the major target of free radicals. Guanine is oxidized to 8-oxyguanosine (8-OHDG), which causes a Guanine - Thymine transverse mutation, perturbing sufficient binding of the sheltering proteins^30^. These oxidatively-modified telomere clusters are less likely to be repaired during the cell cycle and prone to “break” during the next round of replication^31^,^32^. Therefore, telomeres are considered “ROS-traps” that serve to shield the coding parts of the DNA from increased oxidative damage^4^,^7^. We are aware that this post-hoc explanation stems from our purely correlative finding that both telomere lengths and the likelihood of reproduction increased with age, and the assumption that reproduction in dormice is associated with increased oxidative stress. Currently, we cannot rule out that the positive association between age and reproduction may be due to the selective disappearance of certain phenotypes during early adulthood, as found in other species^33^,^34^. Clearly, more experimental work is needed to demonstrate that dormice indeed elongate telomeres in preparation for oxidative stress during reproduction.

The fact that older dormice are able to not only maintain but even lengthen telomeres raises the question why to date this pattern seems rare, or even unique to dormice. One potential trade-off associated with relatively long telomeres, at least in humans, seems to be increased risk of developing cancer^35^,^36^,^37^. However, this appears to be highly dependent on the type of cancer, and long telomeres are even associated with decreased risk of acquiring certain malignant tumours^38^. Also, while telomerase activity is increased in tumour cells, this appears to be merely a consequence of the loss of homeostatic control of cell function, and telomerase itself is not an oncogene^39^. Thus, the evidence for potential drawbacks of maintaining relative long telomeres is ambiguous at best. Moreover, there is evidence that species selected for high longevity, such as the naked mole rat (*Heterocephalus glaber*), increase tumour resistance by up-regulating tumour suppressor barriers (review in de Jesus and Blasco^40^). It would be interesting to test if this is also the case in the long-lived edible dormouse and whether these pathways, together with up-regulated mechanisms of telomere elongation such as telomerase, incur significant increases in metabolic rate. High energetic costs may be another possible trade-off involved in telomere maintenance and lengthening, but to our knowledge, this question remains to be investigated.

However, despite possible trade-offs associated with continuous elongation of telomeres at advanced age, we would be surprised if this pattern is unique. As we attribute our findings to the life-history of *Glis glis*, candidates for age-related telomere elongation include all species that reduce extrinsic mortality, for instance by the ability to escape predation via hibernation^17^ and/or flight^41^,^42^, which selects for high investment into somatic maintenance^43^,^44^. Telomere elongation at older age may also be more likely in species that show no reproductive senescence or even increased probability to reproduce at an older age, as is the case, for example, in some species of bats^45^.

Irrespective of the function underlying gradual telomere elongation in older dormice, and the possible trade-offs involved in this phenomenon, our data clearly refute the assumption that telomeres generally shorten with age in all species (review in Simons^46^). Telomeres may shorten with age in humans and several animal models^46^,^47^, but certainly not in all animals. Hence our findings add to accumulating evidence^46^,^47^,^48^,^49^ challenging the view that telomere length and telomere shortening rates are reliable biomarkers of ageing across species. Our findings clearly reject the notion that there is a universal and inevitable progressive shortening of telomeres that limits the number of remaining cell cycles and predicts longevity.

## Methods

### Capture and Manipulation

130 irregularly positioned nest-boxes (height: 2–3 m) in the Vienna Woods, Austria (48°05′N, 15°54′E, altitude 400–600 m a.s.l.) were checked at fortnightly intervals (for further details about the study site, nest-box distribution and position see Hoelzl *et al*.^50^ and Lebl *et al*.^51^). Animals occupying the nest-boxes (i.e., using nest-boxes as a sleeping site and to rear their young) were captured during the active season (April–October). Newly captured individuals were sexed, weighed to the nearest 2 g using a 300-g spring balance (Pesola®, Baar, Switzerland) and marked with subcutaneous transponders (BackHome BioTec®, Virbac Limited, Bury St. Edmunds, UK; Tierchip Dasmann®, Greven, Germany). Dormice can be reliably classified as juveniles, yearlings (after their first hibernation) and adults (after their second hibernation) from their size, tibia length and fur colour^52^,^53^. Only animals for which an accurate determination of birth year was possible were used in this study. Reproductive activity was determined on a yearly basis. Males were classified as reproductively active when they developed their testes to a tangible and measureable size (for details see Lebl *et al*.^54^). Females were classified as reproductively active when they gave birth to a litter or if they were captured with visible, enlarged mammae. If a female was captured at least twice within the time of young-rearing (week 31–39 of the year, c.f. Lebl *et al*.^51^) without young and/or visible mammae, it was classified as non-reproductive. A total 49 individuals were investigated (7 animals were sampled 5 times, 12 individuals 4 times, 15 individuals 3 times, and 15 individuals 2 times) in this study. The mean timespan between consecutive sampling dates was 178.3 days. At first sampling date 15 individuals were 1 year old, 20 were 2 years, 1 was 3 years, 4 were 4 years, 4 were 5 years, 2 were 6 years, 1 was 7 years and 2 were 8 years old.

The study was approved by the institutional ethics committee (University of Veterinary Medicine, Vienna) and the national authority (Federal Ministry of Science, Research and Economy, BMWF; permit number: BMWF-68.205/0112-II/3b/2011). All experimental protocols were carried out in accordance with the approved guidelines and the Law for Animal Experiments § 8ff, Tierversuchsgesetz – TVG.

### Determination of Relative Telomere Length

To obtain cells for DNA extraction we collected buccal mucosa. This tissue was chosen because it could be obtained using minimally invasive protocols. The alternative, that is, repeated collection of adequate quantities of blood (containing non-nucleated blood cells) is not feasible for free-living edible dormice. Further, numbers and ratios of the different fractions of white blood cells in edible dormice vary within and between seasons^55^, which make this cell type impractical for longitudinal studies. Cell turnover rates of the buccal mucosa cells are relatively constant and unlikely to vary within and between seasons^56^. Buccal mucosa was sampled throughout the active season by twirling Gynobrush® brushes (Heinz Herenz Medizinalbedarf, Hamburg, Germany) on the inner cheek for 15–20 seconds. The heads of the brushes were individually placed into separate 1.7 ml Mμlti®-SafeSeal®Tubes (Carl Roth GmbH+Co. KG, Karlsruhe, Germany) containing 1 ml BC-buffer^57^ and stored at 4 °C for subsequent DNA extraction, which was always carried out within 24 hours after tissue cell collection. Therefore the cells were pelleted and the brushes were removed. The brushes were centrifuged (3000 rpm) for 10 min and the remaining cell pellets (after removal of the brushes) were centrifuged again (5000 rpm) for 2.5 min and 800 μl of the supernatant was discarded. DNA was extracted using a DNeasy Blood&Tissue Kit® (Qiagen) according to the manufacturer’s protocol. Extracted DNA was stored at −20 °C for further analysis.

For measuring Relative Telomere Length (RTL) we used the real-time PCR approach^58^ adapted for edible dormice. As a reference non-variable copy number (non-VCN) gene we used a 54 bp portion of the c-myc proto-oncogene, which was tested for non-variability in copy number in edible dormice by Turbill *et al*.^12^ as described by Smith *et al*.^59^. Primer Sequences for the non-VCN gene were 5′-GAG GGC CAA GTT GGA CAG TG-3′ (c-mycF), and 5′-TTG CGG TTG TTG CTG ATC TG-3′ (c-mycR) and telomeric primer sequences were 5′-CGG TTT GTT TGG GTT TGG GTT TGG GTT TGG GTT TGG GTT-3′ (tel 1b) and 5′-GGC TTG CCT TAC CCT TAC CCT TAC CCT TAC CCT TAC CCT-3′ (tel 2b), respectively. Telomere and non-VCN gene PCRs were carried out in separate runs with 20 ng DNA per reaction, 400 nM of each primer (Tel1b/Tel2b or c-mycF/c-mycR) in a final volume of 20 μl containing 10 μl of SensiMix SYBR No-ROX-MasterMix (Bioline). PCR conditions for the telomere primers were 10 min at 95 °C followed by 40 cycles of 10 sec at 95 °C, 20 sec at 56 °C and 20 sec at 72 °C. For c-myc, PCR conditions were 10 min at 95 °C followed by 40 cycles of 10 sec at 95 °C, 20 sec at 61 °C and 20 sec at 72 °C. A final melting step was included in each run with the temperature ramping from 65 °C to 95 °C in 1 °C steps to check for target specificity via unimodal melt dissociation peaks. All ratios of telomere to non-VCN gene were compared to a reference standard sample (RTL = 1), which was included in every run along with a further calibration standard to monitor inter-run variation. A negative (no-template) control was also included in each run. To validate the low inter run variability of the qPCR approach one run was performed twice (both, qPCR and non-VCN reaction) under the same conditions as outlined above. Furthermore samples of dormice with different ages were evenly assigned to runs to avoid run-to-run bias associated with age. Reactions were prepared using the Qiagility PCR robot (Qiagen, Germany) to minimize pipetting errors and cycling was performed on a Rotorgene Q quantitative thermocycler (Qiagen, Germany). All samples and controls were run in triplicate. We used the software LinRegPCR (2012.0)^60^ for analysis of non-baseline-corrected raw qPCR data, exported from the instrument^59^. RTL was calculated using equation 1, that is, a formula modified for RTL measurements described previously in Ruijter *et al*.^61^ containing RTL = relative telomere length, E = qPCR efficiency, Ct = cycle threshold, T = telomere reaction of target sample, ST = telomere reaction of standard sample, C = control gene (c-Myc) reaction of target sample and SC = control gene reaction of standard sample.


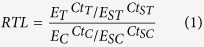


**Figure 5:**
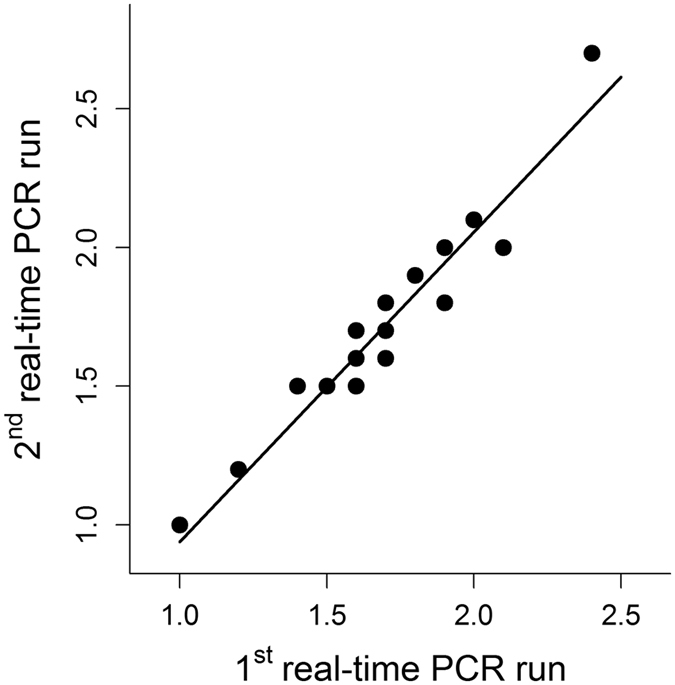
Relative telomere length (RTL) of two qPCR runs with identical samples showing the low inter-run variability of the qPCR method used in this study.

Mean qPCR efficiencies were 94.86% and 96.30% for the non-VCN gene and telomere reactions respectively. The mean coefficient of variation among replicates (intra-assay variation) for Ct-values of the non-VCN gene and telomere assay were 0.020% and 0.016% respectively. Among runs (inter-assay variation) we found a high correlation of RTL (R = 0.96) and a slope (1.12) close to the theoretical optimum of 1.0 between two runs with identical samples (Fig. 5). It should be noted that the inter-assay variation in our study was substantially smaller (≤ 0.02%) than in many other qPCR studies (0.9–7%; review in Steenstrup *et al*.^62^). Therefore, and because there is no logical explanation why errors should depend on the animals ages, we can rule out that measurement error alone could have led to observations of RTL increases, as suggested by Steenstrup *et al*.^62^.

Arguably, the main factors that enabled us to achieve extremely low inter-assay variation were (i) running all qPCRs in a laboratory with controlled constant air temperature. Fluctuations in ambient temperature during the cooling phase of amplification cycles strongly affect the results, as the used qPCR device uses ambient temperature for cooling, which leads to differences in the duration of the different steps (especially primer annealing and elongation). (ii) Additionally we performed a pre-run (run without samples in the instrument) to preheat the thermocycler to avoid temperature fluctuations when several runs were performed in a row. (iii) Furthermore all assays were run with exactly the same reagents and with a strict temporal protocol.

### Statistics

All statistical tests were carried out using R 3.2.1^63^. As RTL were determined at least twice (median 3, range 2–5) in each animal, we used linear mixed effects models (lme; library ‘lme4’^64^ and ‘lmerTest’^65^) that adjust for repeated measures. First, we used an lme model to investigate the effect of sex, age, body mass, reproductive activity and time of the year of taking the DNA sample on RTL in 158 samples from 49 edible dormice. Because data plots gave some indication for nonlinear effects of age, we also entered the square and cube of age as predictors in the full model. As the cubic term was not significant, it was subsequently removed, but quadratic terms were significant and retained. Second, to investigate factors affecting RTL-changes, that is shortening or elongation, we computed an lme model with RTL at time point t as the response variable, and RTL at time t-1 (see below), as well as the above variables entered as fixed effects. This analysis of RTL-changes additionally contained the independent variable time-interval (in days) between subsequent sampling points. Age at time t was used in this model. To correct for repeated measures, animal ID and qPCR plate ID were used as random factors for all models. These random effects were chosen to adjust for repeated measures and possible qPCR plate effects. Year was not used as a random factor as the AICc of the models increased when it was used to correct for effects of year-to-year variation in environmental conditions. Excluding year as a random effect did not notably affect the p-values of the significant terms.

A potential problem in the statistical analysis of changes in RTL over time is the “regression to the mean”, that is, extreme values measured in a subject during a first trial are likely to lie closer to the mean in subsequent trials^66^. Consequently, one would expect stronger apparent shortening of RTL in animals in which initial RTL was high, and vice versa. This effect would also be expected because the relationship between initial RTL and RTL-loss represents a so-called part-whole correlation^67^,^68^. Verhulst *et al*.^66^ have devised a numerical method to correct for these statistical effects. However, it can be shown that RTL values identical to the values obtained by the correction method of Verhulst *et al*.^66^ can be obtained by simply computing the residuals of a linear model of RTL-loss as a function of initial RTL (Supplementary Information). Therefore, we preferred to include initial RTL (i.e. RTL at t-1) as a covariate in the model of RTL changes. This approach has the advantage that, unlike a residual analysis, all predictor variables are entered simultaneously and the correct degrees of freedom are used.

To further separate between-subject and within-subject effects of age we used within-subject centering as outlined in van de Pol & Wright^69^. To differentiate effects we used both the mean age of individuals (capturing the between-subjects effects) and the difference between age at sampling and mean individual age (capturing within-subjects effects) as fixed effect predictors. Our initial analysis (see above) indicated a significantly nonlinear pattern with RTL decreasing in younger and increasing in older dormice. Thus, between-subjects and within-subjects effects could not be meaningfully computed from a single model with all ages pooled together. Also, there was a strong tendency for a continuous interaction between mean age and delta age (t = 1.99. P = 0.050). Therefore we computed separate models four younger and older individuals for all possible points of separation between these groups. This iterative procedure showed that the combined residual sum of squares of these models was minimised when regressions were computed separately for animals with a mean age of ≤5.3 years and >5.3 years, respectively.

To investigate the relationship between age and reproduction, we analysed an extended dataset (years 2006–2014) obtained from the same study population using the same methods as outlined above. This analysis was restricted to females only, because determination of reproductive effort (encountered with young or in lactating state) seemed more reliable than in males (small versus large testes). To see if the yearly probability to reproduce was affected by age we fitted a mixed-effects logistic regression, using age as a fixed predictor and animal-ID nested within study-year as random effects. This model was fitted using function glmmPQL from the R-library MASS^70^. We use n to denote the number of animals investigated and N to indicate the numbers of observations.

## Additional Information

**How to cite this article**: Hoelzl, F. *et al*. Telomeres are elongated in older individuals in a hibernating rodent, the edible dormouse (*Glis glis*). *Sci. Rep.* **6**, 36856; doi: 10.1038/srep36856 (2016).

**Publisher's note:** Springer Nature remains neutral with regard to jurisdictional claims in published maps and institutional affiliations.

## Supplementary Material

Supplementary Information
