# Supplementary material for: Telomeres are elongated in older individuals in a hibernating rodent, the edible dormouse (Glis glis)
Source: Sci Rep. 2016 Nov 24;6:36856. doi: 10.1038/srep36856 (PMC5121655; doi:10.1038/srep36856)
Supplement: Supplementary Information [file srep36856-s1.pdf]

Telomeres are elongated in older individuals in a hibernating rodent, the edible dormouse (*Glis glis*).

Franz Hoelzl[1*], Steve Smith[1], Jessica S. Cornils[1], Denise Aydinonat[1], Claudia Bieber[1], Thomas Ruf[1]

[1] Department of Integrative Biology and Evolution, University of Veterinary Medicine, Vienna, Austria

[*] Correspondence and requests for materials should be addressed to F.H. (email: franz.hoelzl@vetmeduni.ac.at)

Supplementary Information

Verhulst et al. (2013)[1] devised a method to correct for the regression to the mean when comparing baseline telomere length (X1) to follow up measures (X2) by computing a corrected value D. Below, we show an example for the correction of generated random number for X1 and X2 in the notation of R (R Core Team 2015)[2]. R code and output is shown in bold face.

As Verhulst et al. (2013)[1] we use function mvrnorm (Venables & Ripley 2002)[3] to generate correlated (r = 0.7) random numbers

```
samples=20
r=0.7

library('MASS')
data=mvrnorm(n=samples, mu=c(10, 8), Sigma=matrix(c(1, r, r, 1), nrow=2),
empirical=TRUE)
X1=data[, 1]  # standard normal (mu=10, sd=1)
X2=data[, 2]  # standard normal (mu=8, sd=1)
```

Next, we use the equations given by Verhulst et al. (2013)[1] to compute corrected values D. However, compared with Verhulst et al. (2013)[1] we switch variables X1 and X2 in order to obtain negative values of D for decreases in telomere length and positive values of D for increases:

```
rho=(2*r*sd(X1)*sd(X2))/(sd(X1)^2+sd(X2)^2)
D=(X2-mean(X2))-rho*(X1-mean(X1))
```

Alternatively, we can compute a linear model with X2 as the dependent, and X1 as an independent variable, and obtain the model residuals:

```
model=lm(X2~X1)
Resid=residuals(model)
```

To compare results of both approaches, values of D and residuals are combined to a data.frame:

```
result=data.frame(D,Resid)
head(result)

           D       Resid
1  -0.38532184 -0.38532184
2  -0.09824805 -0.09824805
3   1.16125478  1.16125478
4   0.65028182  0.65028182
5  -0.27865166 -0.27865166
6  -0.84631813 -0.84631813
```

This shows that model residuals are identical to the correction suggested by Verhulst et al. (2013)[1]. Therefore, analyzing the effects of further independent variables will lead to virtually identical results: For instance, one may simulate a decrease of telomere length with increasing age:

```
age=runif(samples,1,15) # generate "ages" ranging from 1 to 15 years
X2=X2-0.1*age
```

The following code re-computes D-values and produces the (abbreviated) regression table of D as a function of age:

```
D=(X2-mean(X2))-rho*(X1-mean(X1))
model1=lm(D~age)
summary (model1)

Coefficients:
             Estimate Std. Error t value Pr(>|t|)
(Intercept)   0.78274    0.29714   2.634   0.0168 *
age          -0.11459    0.03634  -3.153   0.0055 **
```

Very similar results can be obtained without computing D, by entering X1 as a covariate:

```
model2=lm(X2~X1+age)
summary(model2)
```

Coefficients:

	Estimate	Std. Error	t value	Pr(>|t|)	
(Intercept)	-1.61447	2.13725	-0.755	0.46035	
X1	0.72738	0.18436	3.945	0.00104	**
age	-0.11670	0.03998	-2.919	0.00957	**

The slight differences between the estimated age effects occur for two reasons:
1) Computed P-values are based on the correct degrees of freedom only in model 2.
2) Only in model 2 all predictors are simultaneously adjusted for each other, which is desirable.

To illustrate the similarity of results from either method using empirical, rather than simulated data, we computed values of D as outlined above using our measurements of RTL at times t-1 as X1 and at times t as X2. Fig. S1 shows a partial regression plot of the effect of age on D, derived from a linear mixed effects model entering the same fixed and random effects (except for initial telomere length) as in our main analysis (c.f. Fig.2).

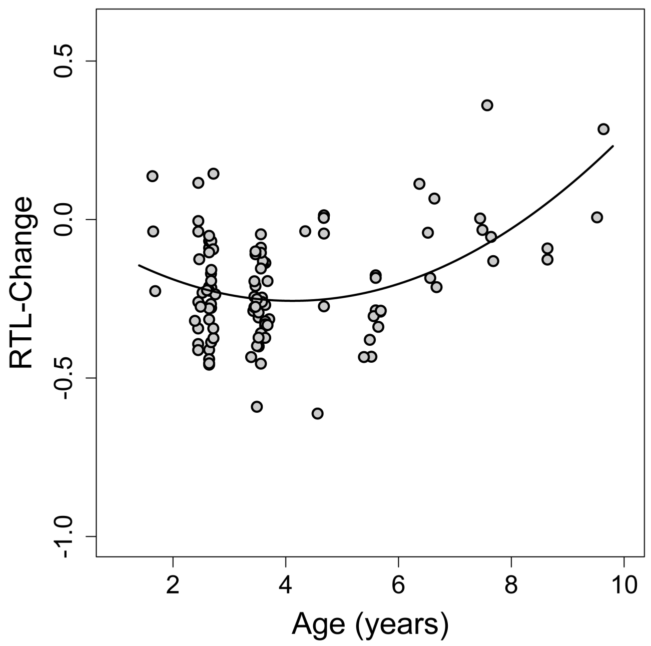

Figure S1. Partial effect of age on RTL-change. RTL-change was computed by correcting differences between subsequent measurements using the method of Verhulst et al. (2013)[1]. Results are almost identical to a model using RTL at time t as the response variable and RTL at time t-1 as a covariate (see Fig. 2).

References

1. Verhulst, S., Aviv, A., Benetos, A., Berenson, G. S. & Kark, J. D. Do leukocyte telomere length dynamics depend on baseline telomere length? An analysis that corrects for 'regression to the mean'. *Eur. J Epidemiol.* **28**, 859-866, doi:10.1007/s10654-013-9845-4 (2013).

2. R: A language and environment for statistical computing. R Foundation for Statistical Computing, Vienna, Austria (2015). <http://www.R-project.org/>.

3. Venables, W. N. & Ripley, B. D. *Modern Applied Statistics with S.* 4th edn (Springer, 2002). <https://www.stats.ox.ac.uk/pub/MASS4/>.
